# Acute Pancreatitis and Prognosticating Its Severity in Young Adults: A Case Report

**DOI:** 10.7759/cureus.22749

**Published:** 2022-03-01

**Authors:** Mallorie Vest, Harneet Grewal, Tanveer Shaukat, Ian Landry, Nso Nso, James O'Connor, Vincent Rizzo

**Affiliations:** 1 Internal Medicine, Icahn School of Medicine at Mount Sinai, NYC (New York City) Health and Hospitals/Queens, New York City, USA; 2 Internal Medicine, St. Georges University School of Medicine, St. Georges, GRD; 3 General Surgery, Icahn School of Medicine at Mount Sinai, NYC (New York City) Health and Hospitals/Queens, New York City, USA

**Keywords:** age, severity, prognostic score, ranson criteria, acute pancreatitis

## Abstract

Acute pancreatitis develops into mild acute, moderately severe, and severe forms in multiple clinical scenarios. The severity assessment of pancreatitis relies on various scoring systems, including CT Severity Index (CTSI), Multiple Organ Dysfunction Syndrome (MODS), Acute Physiology and Chronic Health Evaluation II (APACHE-II), Bedside Index for Severity in Acute Pancreatitis (BISAP), Systemic Inflammatory Response Syndrome (SIRS), Multiple Organ System Score (MOSS), Glasgow score, and Ranson's Criteria (RC). This case report corresponds to a 20-year-old male with acute pancreatitis of unknown etiology. The RC scoring method produced two points, which could not prognosticate the possible severity of acute pancreatitis in the young patient. The hospital course included intubation with mechanical ventilation and ICU management.

## Introduction

The past decades have witnessed an ever-increasing incidence of acute pancreatitis (AP) across the globe. The Atlanta 2012 classification for AP reveals 47.6%, 26.2%, and 26.2% cases with mild, moderately severe, and severe forms, respectively [1]; however, its annual incidence range is recorded as 13-45/100,000 individuals [2]. The mortality determinants in AP cases include sepsis, infection, and pancreatic necrosis. Approximately 1-2% of patients with AP experience mortality [3]; however, the mortality rate in severe AP cases ranges between 20-60% [4]. The contemporary literature reveals a 40% inpatient mortality rate recorded within three days of hospital admissions in the setting of severe AP [5]. In addition, 30-40% of the mortality rate is attributed to hospitalized patients with pancreatitis and organ failure or pancreatic necrosis [6]. The mild AP progresses with minimal fat necrosis and without organ failure or local/systemic complications [7]. The moderately severe AP manifests with transient organ failure (<48 hours) and systemic/local complications; however, patients experience no predisposition to persistent organ failure. In addition, severe AP is characterized by persistent single/multiple organ failure (>48 hours) assessed by Marshall scores obtained from two or more organ systems, including cardiovascular, renal, and respiratory systems [8,9].

The severity assessment in AP cases is paramount to identifying the mortality risk and improving medical management [10]. Research studies indicate 15-20% of AP scenarios with the risk of increased hospital stay duration, severe disease, and mortality [11]. A range of prognostic scoring methods predict AP severity; however, the predominant modalities include Acute Physiology and Chronic Health Examination II (APACHE II) classification and Ranson Criteria (RC) [12]. The other diagnostic approaches include Bedside Index of Severity in Acute Pancreatitis (BISAP), Balthazar CT Severity Index (CTSI), and Glasgow Score (GS) systems [13]. This case report elaborates on the clinical presentation and hospital course of a young adult with AP. It also identifies challenges restricting proper diagnostic assessment and risk stratification in AP cases in the absence of a standardized prognostic scoring system for young patients. 

## Case presentation

A 20-year-old Bengali (Indian) male with no significant past medical history presented to the emergency department with complaints of central abdominal pain for one day. The pain was described as 10 out of 10 in intensity, worsening with movement, and non-radiating in nature. He endorsed shortness of breath upon deep breathing, which subsequently exacerbated the abdominal pain. There were no alleviating factors. He denied any associated nausea, vomiting, fevers, chills, diarrhea, dysuria, or chest pain. His diet has been standard, and he denied the use of medications or supplements at home. He denied use of alcohol, smoking, or illicit drugs. He received two doses of a coronavirus 2019 (COVID-19) vaccine; the second dose was administered three months before admission and the patient denied abdominal pain at that time. Family history was insignificant. On admission, significant labs included a WBC of 26.30/mcL, lipase >3,000 U/L, alanine transaminase (ALT) of 84 U/L, bands of 4.0%, absolute neutrophil count (ANC) of 21.3/mcL, glucose of 142mg/dL, and negative ethanol level. Alkaline phosphatase (ALP), aspartate aminotransferase (AST), and bilirubin were within normal limits. Triglycerides were also within normal limits. IgG4 was 15mg/dL and within the normal range, helping to rule out autoimmune pancreatitis. Hemoglobin A1c (HbA1c) was 5.9%.

**Figure 1:**
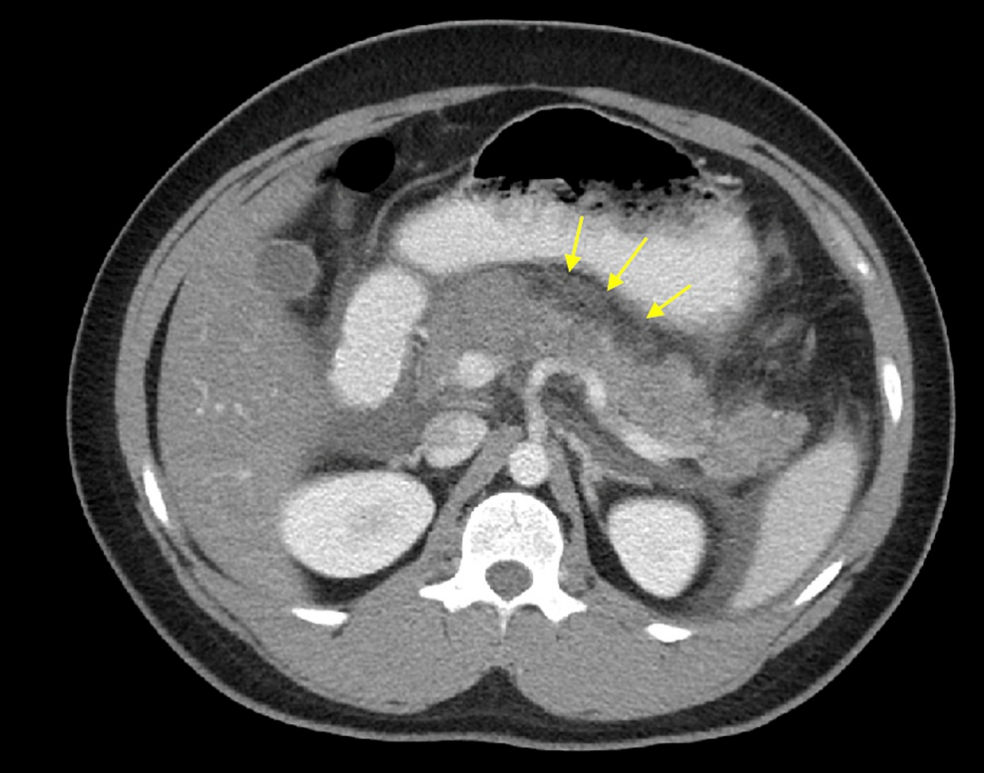
CT of the abdomen/pelvis with contrast showing large amount of fluid surrounding the pancreas (yellow arrows) in the retroperitoneal space, indicating acute pancreatitis. Fatty liver is present.

The patient was admitted to general medical unit with the impression of acute pancreatitis due to an unknown etiology. CT scan of the abdomen/pelvis with contrast showed acute pancreatitis and fatty liver with no evidence of pancreatic necrosis or pseudocyst identified. Gallbladder and bile ducts were normal and no common bile duct (CBD) dilation was noted (Figure 1). A right upper quadrant sonogram revealed trace peripancreatic fluid, hepatic steatosis, small fluid in Morrison’s space, and no evidence of gallbladder disease, cholelithiasis or dilation of the common bile duct. Chest x-ray revealed no evidence of pleural effusions.

**Figure 2:**
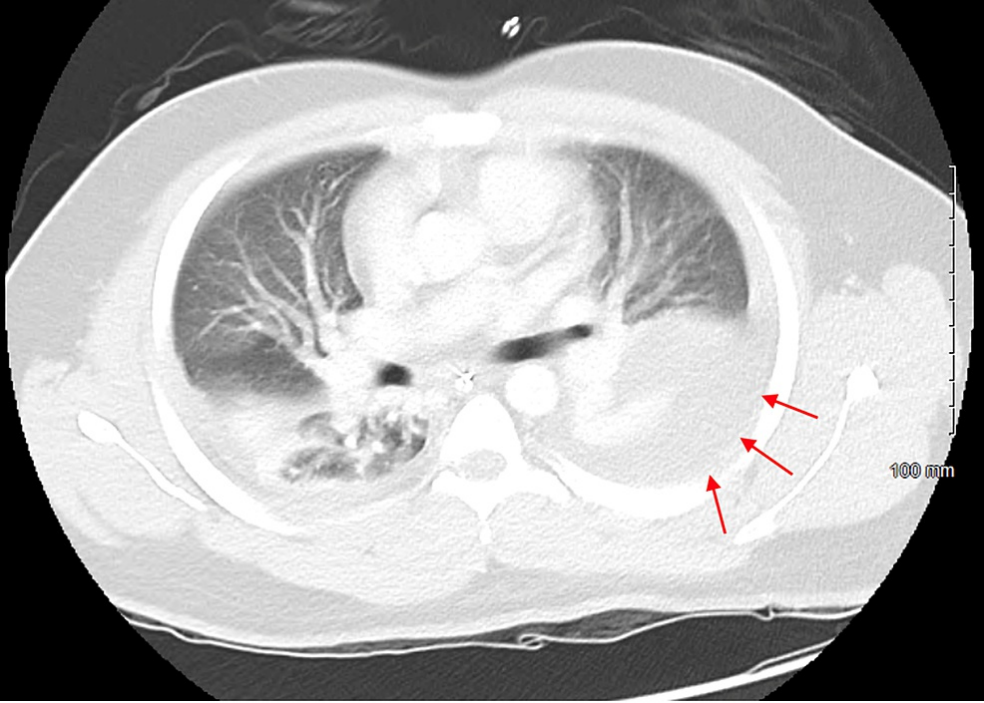
CT chest with contrast showing trace right pleural effusion, large left pleural effusion (red arrows), and bilateral ground glass opacities.

**Figure 3:**
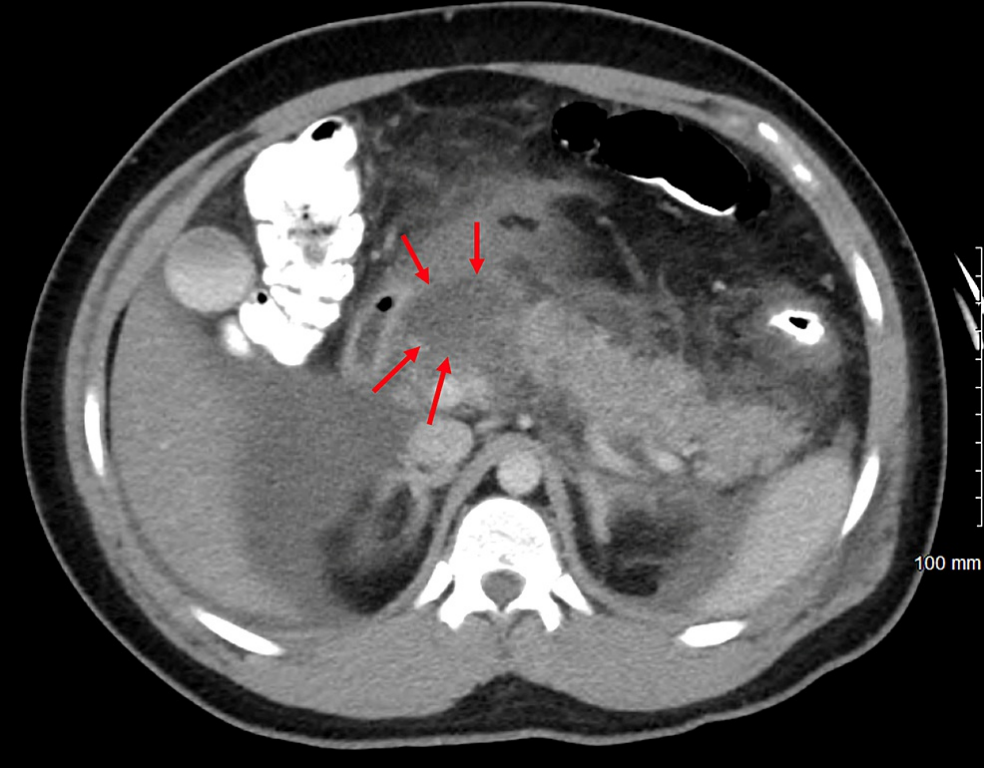
CT abdomen/pelvis with contrast showing fat stranding and fluid around the pancreas with pancreatic necrosis (red arrows).

On hospital day two, the patient developed an increased oxygen requirement and his reported pain remained at 10 out of 10 in intensity. The patient was started on high flow nasal cannula (HFNC) at 40L/min and 40% fraction of inspired oxygen (FiO2). CT scan of the chest with contrast was obtained, which revealed evidence of trace right-sided and moderate to large left-sided pleural effusions, along with bilateral lower lobe compressive atelectasis and bilateral ground-glass appearance of the pulmonary parenchyma (Figure 2). Repeat CT abdomen and pelvis with contrast showed increased fat stranding and fluid around the pancreas, which is suggestive of pancreatic necrosis (Figure 3).

The patient had worsening clinical status including tachycardia at 136 bmp, fever with temperature of 102.2 F, and was upgraded to the step-down unit with the impression of severe necrotizing pancreatitis with acute respiratory distress syndrome (ARDS). The patient developed abdominal distension and a nasogastric tube was placed to suction. Gastroenterology was consulted and recommended broad spectrum antibiotics, IV hydration, IV proton pump inhibitor twice daily, with close surgical follow-up for necrotizing pancreatitis. Infectious disease was also consulted, and the patient was started on meropenem 1-gram IV every eight hours.

The patient remained febrile, tachycardic, tachypneic, and became hypotensive with decreased urine production. He was therefore transferred to the surgical-intensive care unit. A left internal jugular central venous catheter and an arterial line were placed. The patient was intubated and mechanically ventilated on volume control/assist control mode (VC/AC), respiratory rate (RR) of 20, tidal volume (VT) 500 ml, positive end-expiratory pressure (PEEP) 5 cmH2O, and FiO2 70%. Patient was started on dexmedetomidine drip, propofol drip, and fentanyl drip for sedation and pain control.

On hospital day five, the patient had a worsening large left pleural effusion. Interventional radiology placed a left pigtail catheter, which drained yellow serous fluid. The catheter was attached via thora-seal for continuous suction. The patient remained febrile with a WBC of 27.85/mcL. CT abdomen and pelvis with contrast was repeated and again showed necrotizing pancreatitis. It was negative for signs of intraabdominal pancreatic abscess or pseudocyst.

On hospital day nine, the patient remained febrile and had persistent leukocytosis of 27/mcL. Blood cultures remained negative. vancomycin 1g IV every 12 hours and caspofungin 50mg IV daily were started. The patient was awake at this time with weaned sedation of the fentanyl drip. He had abdominal distension with chest x-ray showing dilated loops of bowel and was started on total paraenteral nutrition (TPN).

On hospital day 10, the patient was extubated. After extubation, TPN was discontinued and oral diet was advanced. The patient stated some relief of pain reporting 8 out of 10 in intensity. Leukocytosis improved to 14/mcL and the patient remained afebrile, therefore antibiotics were discontinued. The patient became hemodynamically stable, tolerating room air with an oxygen saturation of 97%. The pigtail chest tube was removed on hospital day 14 without any complication and on day 15, the central venous catheter was removed. The patient additionally denied any pain, nausea, vomiting, or shortness of breath and tolerated a regular diet. The patient was discharged home on hospital day 16. 

The patient has reported to the emergency room multiple times after hospital discharge for recurrent pancreatitis. He is following in gastroenterology clinic for workup of the etiology. He is being worked up for hereditary pancreatitis and is planned to undergo endoscopic ultrasound (EUS) with pancreatic biopsy.

## Discussion

Most patients with AP heal spontaneously after developing mild complications; however, severe forms manifest with early and late phases that often lead to critical complications [14]. Severe AP progresses with necrosis, systemic inflammatory response syndrome (SIRS), and ARDS that potentially elevate the risk for Multiple Organ Dysfunction Syndrome (MODS) [15,16]. The early identification of AP risk factors is paramount to delineating the need for preliminary treatment and ICU management. The evidence-based scoring systems utilize patient-level characteristics for AP assessment; however, prognostic scoring rarely stratifies AP severity in young patients [17]. 

This case study elaborated on the AP progression and prognosis in a 20-year-old patient. RC was utilized to prognosticate AP severity within the initial 48 hours after admission (Table 1) [18]. RC conventionally examines five parameters at admission and six within the subsequent 48 hours. Mild AP is identified by an RC score ranging between 1-3; however, the mortality rate substantially increases with high scores (>4-6) [19]. The RC scores <3, >3, and >6 indicate the risk of 0-3%, 11-15%, and 40% mortality, respectively. Severe AP increases the mortality risk to 36-50%; however, the overall death rate ranges between 3-10%. The patient in the present case had a risk of mild AP based on the reported WBC (26.3/mcL) and lactate dehydrogenase (LDH) (484 U/L) levels that produced 2 points via RC scoring. However, RC proved to be a poor prognosticator of AP since the patient’s spontaneous decompensation triggered the need for ICU management. 

**Table 1:** Ranson criteria LDH: lactate dehydrogenase; AST: aspartate aminotransferase; BUN: blood urea nitrogen

Timing	Prognostic Factor	Score
At admission	Age > 55 years old	1 point
	WBC > 16,000/microL	1 point
	Glucose >200 mg/dL	1 point
	LDH > 350 international units/L	1 point
	AST > 250 international units/L	1 point
At 48 hours	Hematocrit fall of 10% or greater	1 point
	BUN rise of 5 mg/dL	1 point
	Serum calcium <8 mg/dL	1 point
	pO_2_ <60 mmHg	1 point
	Base deficit >4 mEq/L	1 point
	Fluid sequestration >6000 mL	1 point

The currently practiced prognostication methods do not precisely stratify clinical outcomes in young patients with AP. GS and RC systems predict AP severity within 48 hours after admission; however, SIRS, Multiple Organ System Score (MOSS), APACHE-II, and BISAP scores guide the severity assessment within a day of admitting the patient [4]. The five parameters of RC guide the prognostication of AP in patients within the range of 55-70 years. The overall RC scores (0-11) facilitate the severity assessment in patients within the age range of 30-70 years. In addition, 0-2, 3-4, 5-6, and 7-11 RC points indicate the mortality rates of 0-3%, 15%, 40%, and 100%, respectively [12]. Blamey et al. elaborated on the utilization of GS for stratifying the severity risk in AP cases [20]; however, the retrospective study by Kiat et al. confirmed higher sensitivity, negative predictive value, and receiver-operative curve of RS compared to GS for AP prognostication [21]. These findings were limited to patients with an average age of 58.7±17.4 years and, therefore, do not effectively apply to young adults diagnosed with AP. 

A prospective study by Khanna et al. comparatively analyzed AP prognostication tools including CTSI, GS, RC, SIRS, MOSS, APACHE-II, and BISAP; however, the validity of these findings are also limited to patients with a mean age of 40.5 years due to the lower percentage (i.e., 12.5%) of young patients from the age group of 11-20 years [4]. In addition, the retrospective study by Lautz et al. emphasized the incapacity of pediatric acute pancreatitis severity (PAPS), GS, and RC scoring tools to prognosticate AP severity in pediatric patients (≤ 18 years) [22].

## Conclusions

The low RC score in the present case indicated the young patient’s minimal risk for severe AP progression. The need for mechanical ventilation and ICU management on day four of admission, however, confirmed the incapacity of the RC system to predict pancreatitis severity in young patients. In addition, findings from this case presentation and contemporary literature confirmed the poor prognostication of AP severity in young adults by the currently implemented multifactorial scoring approaches. 
